# The role of systemic inflammation in patients with conjunctivochalasis

**DOI:** 10.1007/s00417-025-07101-3

**Published:** 2026-01-09

**Authors:** Mine Koru Toprak, Aydin Toprak

**Affiliations:** 1 Department of Ophthalmology, Giresun Prof. Dr. İlhan Özdemir State Hospital, Giresun, Turkey; 2 Department of Ophthalmology, Giresun University Training and Research Hospital, Giresun, Turkey

**Keywords:** Conjunctivochalasis, Systemic inflammation, Inflammatory biomarkers, Complete blood count (CBC)

## Abstract

**Purpose:**

To compare systemic inflammation parameters and blood lipid profile in patients with conjunctivochalasis versus healthy individuals.

**Methods:**

Thirty patients with conjunctivochalasis(Cch) and thirty age-matched healthy controls were included in this retrospective study. Complete blood count (CBC) parameters and serum levels of total cholesterol, low-density lipoprotein (LDL), high-density lipoprotein (HDL), and triglycerides (TG) were obtained from electronic medical records. The neutrophil-to-lymphocyte ratio (NLR), platelet-to-lymphocyte ratio (PLR) and monocyte-to-HDL ratio (MHR) were calculated for each participant. Group differences were analyzed statistically.

**Results:**

The monocyte-to-HDL ratio was significantly higher in the conjunctivochalasis group (*p* = 0.023), while hemoglobin levels were significantly lower (*p* = 0.001). No additional significant differences were observed in other hematological or lipid parameters.

**Conclusion:**

This study is the first to demonstrate an elevated monocyte-to-HDL ratio in patients with conjunctivochalasis. The concurrent reduction in hemoglobin levels provides further support for a potential systemic association. These findings suggest that conjunctivochalasis may involve not only localized ocular changes but also systemic inflammatory processes.

## Key Messages

*****What is known***:**

- Conjunctivochalasis is associated with age-related ocular surface changes and localized inflammation.

*****What is new***:**

- This is the first study demonstrating significantly increased monocyte-to-HDL ratio (MHR) in patients with conjunctivochalasis.
- Hemoglobin levels were significantly lower in conjunctivochalasis patients compared with controls.
- These findings suggest a potential systemic inflammatory component in the pathogenesis of conjunctivochalasis.

## Introduction

Conjunctivochalasis (CCh) is a frequently encountered age related condition affecting the ocular surface, characterized by the presence of loose, redundant conjunctival folds without accompanying edema and primarily localized in the lower eyelid region. It is considered a significant mechanical alteration associated with ocular aging and its prevalence has been reported to increase substantially in individuals over the age of 60, reaching up to 90% in certain populations [1]. This condition may interfere with tear film stability, disrupt the normal contour of the inferior tear meniscus and contribute to inflammatory responses on the ocular surface. As a result, patients may present with clinical complaints such as ocular dryness, excessive tearing, a sensation of a foreign body and fluctuating or reduced visual clarity [2].

In recent years, various hypotheses have been proposed regarding the pathogenesis of CCh [3]. The predominant pathological findings consist of inflammatory cell infiltration accompanied by elastic fiber degradation in the excessive conjunctival tissue [4, 5]. Furthermore, evidence of oxidative damage has been documented in conjunctivochalasis [6]. Although age-related changes and localized inflammatory responses have been implicated as contributing factors, systemic mechanisms such as systemic inflammation and oxidative stress may also play a role in its progression.

The accessibility and cost-efficiency of common laboratory assessments, including complete blood count (CBC) and standard biochemical analyses, have significantly enhanced the identification of systemic inflammatory states [7]. Furthermore, these routine diagnostic methods provide data that can be used to derive innovative, composite biomarkers of systemic inflammation such as the platelet-to-lymphocyte ratio (PLR), the neutrophil-to-lymphocyte ratio (NLR) and the monocyte-to-high-density lipoprotein ratio (MHR) [8]. Deviations in the concentrations of these biomarkers can indicate an inflammatory imbalance, potentially playing a role in the pathological progression of various ocular conditions including glaucoma and non-arteritic anterior ischemic optic neuropathy [9, 10]. We found that systemic inflammatory factors in the pathophysiology of conjunctivochalasis have not been investigated in the literature. Therefore, in our study, we aimed to compare the complete blood count and biochemical values ​​of patients diagnosed with conjunctivochalasis with those of healthy individuals.

## Methods

This retrospective study was conducted in the ophthalmology clinic of Osmaniye State Hospital. Ethical approval for the study was obtained from the Non-Interventional Clinical Research Ethics Committee of Hatay Mustafa Kemal University (Approval date: 09/07/2024, Decision No: 04). The research strictly adhered to the ethical principles outlined in the Declaration of Helsinki.

The electronic health records of individuals diagnosed with conjunctivochalasis between January 2023 and April 2024 were retrospectively reviewed. Individuals classified as lid-parallel conjunctival folds (LIPCOF) grade 3 and who had documented complete blood count and biochemical test results within the month before their eye examination were included in the study. Systemic comorbidities were screened through a detailed review of each participant’s electronic medical records, including documented medical diagnoses, medication history and available laboratory findings. Exclusion criteria included the presence of additional ocular diseases (such as pterygium), known systemic conditions such as diabetes, hypertension or autoimmune disorders, documented systemic infections within the last month, any history of smoking, or a history of systemic anti-inflammatory, lipid-lowering medications use. Information regarding recent medication use was obtained from electronic medical records. In parallel, a control group of thirty healthy individuals, matched for age and gender, was established.

### Conjunctivochalasis assessment

Medical records of 30 patients diagnosed with grade 3 conjunctivochalasis based on LIPCOF classification were retrospectively reviewed. The severity of conjunctivochalasis was assessed and categorized based on LIPCOF classification system, a concept originally described by Höh et al. which defines the grading as follows [11];

- Grade 0: No visible conjunctival folds are present.
- Grade 1: Single fold become apparent and remain situated below the tear meniscus level.
- Grade 2: Spontaneously visible folds are observed, which are located beneath the tear meniscus.
- Grade 3: Prominent folds extending beyond the tear meniscus height, which were discernible without any blinking stimulus.

### Hematological and biochemical analyses

To be eligible for inclusion, patients were required to have undergone complete blood count and biochemical testing within one month before their eye examination. Relevant laboratory data were retrospectively retrieved from institutional electronic health records. All blood samples, including biochemical and lipid profile measurements, were obtained after a minimum of 12 h of fasting. Hematological parameters including erythrocyte, leukocyte, neutrophil, monocyte, lymphocyte and thrombocyte counts were determined using a Sysmex SP-10 automated hematology system (manufactured by Sysmex Corporation in Kobe, Japan). For serum lipid profiling; HDL, LDL, triglycerides and total cholesterol were conducted using a Cobas e602 chemistry analyzer (Roche Diagnostics, Mannheim, Germany). Based on these data, inflammatory indices such as NLR, PLR and MHR were calculated manually.

### Statistical analysis

All statistical analyses were conducted using IBM SPSS Statistics software, version 25.0 (IBM Corp., Armonk, NY, USA). The normality of continuous variables was evaluated using the Kolmogorov–Smirnov test. Hemoglobin, lymphocyte count, platelet count, mean platelet volume, white blood cell count, monocyte count and LDL values demonstrated normal distribution; therefore, group comparisons for these variables were conducted using the independent samples t-test. In contrast, the distributions of MHR, NLR, PLR, neutrophil count, hematocrit, platelet distribution width, HDL, triglycerides and total cholesterol deviated from normality and were compared using the Mann–Whitney U test. Descriptive statistics were presented as mean ± standard deviation for normally distributed variables and as median (min–max) for non-normally distributed variables. A two-tailed p-value of < 0.05 was deemed to indicate statistical significance.

## Results

Between January 2023 - April 2024, retrospective screening was conducted on 120 individuals initially diagnosed with conjunctivochalasis. Of these, 106 had accessible results for both blood and lipid profiles obtained within the prior month. Seventy patients were excluded due to systemic comorbidities. An additional six were removed based on the presence of ocular conditions such as pterygium, dry eye, glaucoma, or macular degeneration. Following this filtration, 30 conjunctivochalasis patients meeting all inclusion criteria were included in the study, alongside a matched control group of 30 healthy individuals with no systemic or ocular disease history.

The conjunctivochalasis group comprised 14 females (46.7%) and 16 males (53.3%), while the control group consisted of 13 females (43.3%) and 17 males (56.7%) (*p* = 0.507). The average age in the conjunctivochalasis cohort was 69.70 ± 6.39 years, closely comparable to the control group’s average age of 69.90 ± 5.6 years (*p* = 0.898). The demographic characteristics of both groups are presented in Table 1.

**Table 1:** Comparison of demographic characteristics between patients with conjunctivochalasis and healthy controls. *p* < 0.05 was considered statistically significant

	Patients (*n* = 30)	Control (*n* = 30)	*p* value
Age (years), Mean ± SD	69.70 ± 6.39	69.90 ± 5.60	0.898
Sex, n (%)			0.795
Male	16 (53.3%)	17 (56.7%)	
Female	14 (46.7%)	13 (43.3%)	

Our analysis demonstrated that monocyte-to-HDL cholesterol ratio (MHR) was observed to be markedly elevated in patients diagnosed with conjunctivochalasis in comparison to the control individuals (U = 233.0, Z = − 3.22, *p* = 0.001; *r* = 0.42), as shown in Table 2. Conversely, the hemoglobin concentrations exhibited a significant decrease within the conjunctivochalasis cohort when compared to healthy control participants (95% CI: -1.34 to − 0.11; *p* = 0.023). Other complete blood count parameters, specifically lymphocyte counts, platelet levels, mean platelet volume (MPV), white blood cell counts, monocyte levels, neutrophil counts, hematocrit and platelet distribution width, did not reveal any statistically meaningful differences between the study groups. Likewise, the components of the lipid profile, including LDL, total cholesterol, HDL and triglycerides, showed no statistically significant variations across the groups (Table 3).

**Table 2:** Complete blood count and systemic inflammatory index parameters of patients with conjunctivochalasis and control group (PLR, platelet-to-lymphocyte ratio; NLR, neutrophil-to-lymphocyte ratio; MHR, monocyte-to-high-density lipoprotein ratio). Values are presented as mean ± standard deviation. *p* < 0.05 was considered statistically significant

	Patients (*n*:30)	Controls (*n*: 30)	*P* value
	Mean ± SD	Mean ± SD	(Student’s t test)
White blood cell count (x109/l)	7.43 ± 1.03	7.73 ± 1.68	0.4
Lymphocyte count (x109/l)	2.38 ± 0.44	2.43 ± 0.47	0.677
Monocyte count (x109/ l)	0.54 ± 0.08	0.58 ± 0.13	0.208
Platelet count (x109/l)	267 ± 43.32	268 ± 65.95	0.945
Mean platelet volume (fl.)	10.5 ± 1.22	10.58 ± 0.95	0.779
Hemoglobin (g/dl)	13 ± 1.13	13.73 ± 1.25	**0.023**
PLR	116.4 ± 31.6	114.39 ± 35	0.816
	Median (Min–Max)	Median (Min–Max)	p value
			(Mann–Whitney U test)
Neutrophil count (x109/l)	3.86(2.5–5.9)	4.056(2.4–7.2)	0.408
Platelet distribution width (%)	12.87(9.4–20.7)	12.44(8.3–18.6)	0.636
Hematocrit (%)	39.3(34.1–45.7)	40.92(34.8–46.4)	0.086
NLR	1.68(1.1–2.5)	1.73(0.9-4)	0.865
MHR	0.051(0.005–1.01)	0.012(0.004–0.021)	**0.001**

**Table 3:** Lipid profile parameters in patients with conjunctivochalasis and control group (LDL, low-density lipoprotein; HDL, high-density lipoprotein; ). Values are presented as median (min–max). *p* < 0.05 was considered statistically significant

	Patients (*n*:30)	Controls (*n*: 30)	*P* value
	Mean ± SD	Mean ± SD	(Student’s t test)
LDL (mg/dl)	112.2 ± 18.1	115.05 ± 23.6	0.602
	Median (Min–Max)	Median (Min–Max)	p value
			(Mann–Whitney U test)
Total cholesterol (mg/dl)	195.03(146.6-239.3)	196.64(142-302.4)	0.482
HDL (mg/dl)	51.58(39.5–73)	49.39(30.5–85)	0.225
Triglyceride (mg/dl)	146(51-361.4)	143.5(67–285)	0.684

Post-hoc effect size analyses supported the robustness of these findings with a moderate-to-strong effect size for MHR (rank-biserial correlation = 0.45) and a moderate effect size for hemoglobin (Cohen’s d = 0.60) despite the modest sample size.

## Discussion

To date, studies investigating conjunctivochalasis have predominantly focused on local ocular surface mechanisms. To the best of our knowledge, this is the first study to evaluate systemic inflammatory indices in patients with conjunctivochalasis. Our findings showed that the monocyte-to-HDL ratio (MHR) was higher and hemoglobin levels were lower in the conjunctivochalasis group. These results suggest that conjunctivochalasis might be associated with systemic conditions.

Previous studies investigating the pathology of conjunctivochalasis have predominantly emphasized local inflammation and elastotic degeneration [6]. Two different reports demonstrated increased levels of inflammatory mediators such as TNF-α, IL-1β, IL-6, IL-8 and IL-12 in the tear film of patients with conjunctivochalasis [6, 12]. Similarly, Fodor et al. reported diffuse conjunctival inflammation in advanced cases, resembling the inflammatory patterns observed in dry eye disease [13]. Another study noted that a subset of conjunctivochalasis specimens exhibited chronic non-granulomatous inflammation or elastosis, indicating that both mechanisms may contribute to disease development [14]. In a separate histopathological investigation, Harbiyeli et al. observed increased inflammation, elastic fiber damage and goblet cell loss in conjunctival tissue samples [15].

Beyond these local mechanisms, hematological inflammatory indices such as NLR, PLR and MHR have emerged as practical markers of systemic inflammation, particularly in rheumatologic conditions [16]. While monocytes play a central role in the secretion of pro-inflammatory cytokines, HDL exerts antioxidant, anti-inflammatory and endothelial-protective effects [17]. Thus, an elevated MHR is considered a sensitive indicator of systemic inflammatory burden [18]. Increased systemic inflammatory ratios have also been reported in ophthalmic disorders including keratoconus, non-arteritic anterior ischemic optic neuropathy and glaucoma [9, 10, 19]. Satirtay et al. further demonstrated significantly higher MHR levels in patients with branch retinal vein occlusion [20]. In our study, MHR values were similarly elevated in the conjunctivochalasis group, suggesting a potential association between conjunctivochalasis and systemic inflammatory status. However, it should be emphasized that MHR is a nonspecific marker of systemic inflammation and may be influenced by unmeasured systemic factors. Therefore, this finding should be interpreted as an association rather than a causal relationship.

Hemoglobin is essential for efficient oxygen delivery to tissues and low levels trigger oxidative stress in tissues [21]. Ward et al. showed increased lipid and DNA oxidative stress in conjunctival chalazion specimens and suggested that inflammatory and collagenolytic changes may contribute to conjunctival laxity [6]. Wu et al. have shown that hypoxia play a role in scleral remodeling during myopia progression through the activation of hypoxia-induced factors (HIFs), which regulate extracellular matrix synthesis and may contribute to axial elongation [22]. Although lower hemoglobin levels in our conjunctivochalasis group may biologically contribute to increased oxidative stress in conjunctival tissues, larger prospective studies are needed to confirm this association.

From a clinical perspective, our findings suggest that adopting a more comprehensive evaluation including consideration of systemic indicators such as hematologic parameters may be beneficial in the assessment of patients with conjunctivochalasis. Moreover, our results raise the possibility of personalized therapeutic strategies including the potential use of systemic anti-inflammatory approaches. Nevertheless, larger prospective studies are required to clarify the nature and clinical relevance of this relationship.

This study has several limitations. First, its retrospective design and the relatively small sample size introduce a risk of selection bias and limit the strength of the conclusions. Furthermore, histopathological evaluations were not included. Additionally, systemic inflammatory biomarkers such as CRP and IL-6 were not measured, making it difficult to fully characterize the systemic inflammatory profile of these patients. Finally, multivariate analysis could not be performed due to sample size constraints. Prospective studies with a larger patient population including detailed inflammatory markers and histopathological evaluations are necessary to strengthen the validity of our results.

In summary, although previous studies have emphasized the role of local inflammatory mechanisms in conjunctivochalasis, our findings suggest that the condition may not be confined solely to the ocular surface but could be associated with broader systemic processes. From this perspective, evaluating conjunctivochalasis within a more comprehensive systemic framework may be beneficial, and further studies are warranted to clarify this potential relationship.
